# Data on population density, growth, survival, water quality, larval stage index and ingestion rate of selected microalgae of portunids crabs at different feeding regimes

**DOI:** 10.1016/j.dib.2019.104438

**Published:** 2019-08-28

**Authors:** Muhammad Nur Syafaat, Taufik Muhammad, Ambok Bolong Abol-Munafi, Mhd Ikhwanuddin

**Affiliations:** a Research Institute for Brackishwater Aquaculture and Fisheries Extension (RIBAFE), Maros, 90512, South Sulawesi, Indonesia; b Institute of Tropical Aquaculture and Fisheries Research (AKUATROP), University Malaysia Terengganu, Kuala Nerus, 21030, Terengganu, Malaysia; c STU-UMT Joint Shellfish Research Laboratory, Shantou University, Guangdong, 515063, China

**Keywords:** Aquaculture, Crablet, Development, Growth, Larval rearing, *Scylla tranquebarica*, *Portunus pelagicus*, Microalgae selection

## Abstract

Population density, growth, survival, water quality and larval stage index of purple mud crab, *Scylla tranquebarica* at different feeding regimes and the data on ingestion rate of chosen microalgae, survival and larval development of blue swimming crab, *Portunus pelagicus* are presented. A twenty days of *S. tranquebarica* larval culture from zoeal 1 until megalopa stage under two different feeding regimes of A) Rotifer, *Artemia* nauplii and shrimp meat and B) Rotifer, *Artemia* nauplii and artificial feed is shared. A method on investigation of individual larvae of *P. pelagicus* capability to catch four different types of microalgae within 24 h is also shared. Direct eye observation, data collected through the larval rearing culture of *S. tranquebarica* and further statistical analysis were done daily until the crabs reached the megalopa stage. The result obtained from the optimum density of selected microalgae fed by individual larvae of *P. pelagicus* will be combined with the highest survival rate and larval stage index to develop feeding schedule for crab larvae *P. pelagicus*. This dataset has not previously been published and is of great potential for further comparison with other – and future investigation of various feeding regimes affected the crab culture. The collected information could be used as a standard feeding regime for nursery and hatchery seed production of others portunids crabs. The data described in this article are available as a supplementary file to this article.

**Table:** Specifications Table

Subject	Agriculture and Biological Sciences; Aquatic Sciences
Specific subject area	Aquaculture Nutrition and Larval Development
Type of data	Table, Chart and Graph
How data were acquired	Direct observation, daily data recorded through hatchery works, titration method, larval rearing, particle counter machine
Data format	Raw, Analyzed and Filtered
Parameters for data collection	For Mud Crab Data: Larval Stage Index (LSI), Percentage of Megalopa Occurrence Index (MOI), Daily population density (ind/L), Survival rate (survival rate from Zoea 1 to Zoea 5 and M, survival rate from Zoea-1 to crablet and survival rate from M to crablet), Crablet production (the number of crablets produced at the end of the study for each treatment), Mean body weight, carapace length and carapace width of the crablet produced, Water quality includes. The Total Ammonia Nitrogen (TAN), Nitrite (NO_2_), Total Organic Matter (TOM) and Total Bacteria of *Vibrios* (TBV) were also recorded. For Blue Swimming Crab: Calculate before and after microalgae density using Particle counter machine for ingestion rate experiment. Taking weight of each larvae stage using analytical balance at the end of day culture (14 days) and make classification on morphology of larvae stage using compound microscope along culture period
Description of data collection	LSI: daily = ((A1xA2)+(B1xB2))/C, A1 = amount of previous stage larvae, A2 = previous stage, B1 = amount of highest stage larvae, B2 = highest stage, C = total amount of sample; MOI: based on the occurrences of M per 100 individual Zoea-5; population density: counting the number of larvae in 100 ml of culture media; crablet production: the number of crablets produced at the end of the study; Microalgae consuming after 24 h by individual larvae according to their larval stage
Data source location	Institute of Tropical Aquaculture and Fisheries Research (AKUATROP), Universiti Malaysia Terengganu, 21030, Kuala Nerus, Terengganu, Malaysia and Research Installation of Marana belongs to Research Institute for Brackishwater Aquaculture and Fisheries Extension (RIBAFE), Maros, South Sulawesi, Indonesia
Data accessibility	With the article

**Table:** 

**Value of the data** • Recently, mud crab genus *Scylla* has been used as the targeted species for aquaculture commercialization [1], [2]. Thus, data of this article increase the information related to the mud crab larval culture especially when they fed at various feeding protocols. • Generally, the gut content of *P. pelagicus* larvae are consist of algal filament, fragment of benthic organism (diatoms, polychaetes, nematodes, foraminiferans and radiolarians) [3], [4], [5] therefore for establishing the suitable feeding, it is compulsory to investigate the effects of different microalgae as feed for *P. pelagicus* larvae. • The data can be used by hatchery manager to conduct the proper feeding protocols and feeding schedule of portunids crab. • Studies on different microalgae preference on *P. pelagicus* larvae at different stages are critical to understand when and how much they impact specifically on survival and growth development [6]. • The significant to determine optimum density of various microalgae consumed by individual *P. pelagicus* larvae, its gives additional knowledge about the amount food consumed by crab larvae, which can reduce the mass mortality of larvae during rearing [7]. • The raw data obtained from the different feeding regimes experiment will allow other researcher to develop the suitable feeding regimes for the portunids crabs crablets production. • Data presented here could be useful possible future feed formulation of portunids crabs [6], [8]. • This data also can be used for possible collaboration with other research institution as well as comparison to other environmental conditions in portunids crab [9], [10], [11].

## 1 Data

The article includes the data on population density, growth, survival, and larval stage index of purple mud crab, *Scylla tranquebarica* culture at different feeding regimes. Each data was categorized in different sheets of .xlsx file. In addition, water quality which includes Total Ammonia Nitrogen (TAN), Nitrite (NO_2_), Total Organic Matter (TOM) and Total Bacteria of *Vibrios* (TBV) were also recorded during the feeding regimes experiments (.xlsx). This dataset also provides detailed information on mean ingestion rate of four different microalgae used by individual crab larvae after 24 hour for Z1 till Z4, mean daily percentage survival (%) of *P. pelagicus* larvae fed with selected microalgae and mean number of Larvae Stage Index (LSI) of *P. pelagicus* larvae fed with selected microalgae.

## 2 Experimental design, materials, and methods

### 2.1 Different feeding regimes experiment

This study on *S. tranquebarica* was conducted at Research Installation of Marana belongs to Research Institute for Brackishwater Aquaculture and Fisheries Extension (RIBAFE), Maros, South Sulawesi, Indonesia and Institute of Tropical Aquaculture and Fisheries Research (AKUATROP), Universiti Malaysia Terengganu, Terengganu, Malaysia. This study focus on feeding strategy used two feeding regimes namely Treatment A (without artificial feed) and Treatment B (with artificial feed) each with 3 replications (Table 1).

**Table 1:** Feeding regime of purple mud crab, *Scylla tranquebarica* larvae rearing with and without artificial feed.

STAGES	A			B		
	Rotifer (ind/ml)	*Artemia* nauplii (ind/ml)	Shrimp meat (*Metapenaeus* spp) (ppm or g/m^3^)	Rotifer (ind/ml)	*Artemia* nauplii (ind/ml)	Artificial feed (ppm or g/m^3^)
Zoea 1	20–40			20–40		
Zoea 2	20–40			20–40		
Zoea 3	10	0.5		10	0.5	0.5
Zoea 4	10	1		10	1	0.7
Zoea 5	–	2		–	2	1
Megalopa	–	3–5	2	–	3–5	2
Crablet	–	1–5	1–5	–	1–5	1–5

#### 2.1.1 Water sources

This study used the underground saline water (salinity 26–32 ppt). Pumped water stored in a concrete tank which is also serves as a sedimentary tank. The water then chlorinated (with Calcium hypochlorite powder - available chlorine 60%) at 20 ppm overnight before neutralized with sodium thiosulfate at 10 ppm. Gentle aeration was provided in the reservoir to promote chlorine ionization. After the water in the initial tank becomes neutral, the water was moved into the second reservoir through a sand charcoal filter tank. The water in the second reservoir tank is then drained into the third reservoir through membrane filter (0.01 μm), zeolite, and ozonation tubes. Before drained to the larvae rearing tank, the water in the third reservoir was drained through an ultra violet light (36 W, Phillips®).

#### 2.1.2 Larval rearing

Larvae rearing from Zoea-1 (Z1) up to Zoea-5 (Z5) used conical cylindrical fiber glass tank with a volume of 200 L at an initial density of 50 ind./L^−1^. Thinning was done when the larvae entering the megalopa (M) stage by moving the megalopa to a larger rearing tank. Rearing tank for M stage was a round fiber tank (volume ±4 tons) and stocked with a density 1.5 ind/L^−1^. Rearing of *S. tranquebarica* larvae begins at Z1 to 3rd crablet stage (C3). New hatched larvae collected in a basin and given Elbayou (powder form which contain 100 mg Nifustyrenate-Sodium/1 g as a bacterial treatment, produced by Ueno food techno industry, Ltd.) as much as 5 mg/L and then larvae stocked in the conical tanks, then counted larvae density in each conical tank. Erythromycin was applied into the rearing medium as much as 3 ppm at the beginning of the experiment and at each change of larvae stage. Treflan® also applied once a week into the rearing water at a dose of 0.05 ppm. Water exchange performed every two days started at the sevent days of larvae culture at a rate of 10–20%. During the culture period, the photoperiod system was applied used nature ligth with indoor condition. The larval rearing culture techniques were followed the previous published methods for portunids crabs [12], [13].

#### 2.1.3 Feeding regime

A commercial feed for shrimp larvae (PL2-PL5) (Frippak® PL+150) with a size of 100–200 μm was used and applied twice a day at 0900 hours and 1600 hours from Z3 stage to C3 stage. Fresh feed used for M and crablet phase was a crumbled shrimp-meat applied twice a day at 0800–1000 hours and 1400–1600 hours. Rotifer (*Brachionus plicatilis*) and *Artemia* nauplii (Mackay Marine *Artemia*® (green can)) were enriched in advance with HUFA (*High Unsaturated Fatty Acid*) (A1-DHA SELCO INVE®, contains n-3 HUFA = 200 mg/g dry weight with ratio of DHA/EPA = 2,5) at doses of 40 and 80 ppm sequentially with duration of about 1–2 hours for rotifers and 2–3 hours for *Artemia* nauplii. The dosage and type of feed given for each treatment was shown in Table 1.

#### 2.1.4 Parameter

##### 2.1.4.1 Larval stage index (*LSI*)

LSI was calculated as daily by using equation, LSI = ((A1xA2)+(B1xB2))/C, A1 = amount of previous stage larvae, A2 = previous stage, B1 = amount of highest stage larvae, B2 = highest stage, C = total amount of sample. Larvae population was observed every day starting at 2 Days After Hatching (2 DAH) until the 20 DAH by taking samples of larvae as many as 10 ind.tank^−1^. To calculate the LSI in each treatment, the scoring technique was applied to each larvae stage, namely; Zoea-1 = 1, Zoea-2 = 2, Zoea-3 = 3, Zoea-4 = 4, Zoea-5 = 5, Megalopa (M) = 6. For example, from the 10 larvae sampled, three larvae in Zoea-1 stage; two larvae in Zoea-2 stage and five larvae in Zoea 3 stage are found. The LSI value then calculated using the following equation:

LSI=(3x1+2x2+5x3)/10=2.2

##### 2.1.4.2 Percentage of megalopa occurrence index (MOI)

The MOI value was calculated based on the occurrences of M per 100 individual Zoea-5 [14], [15]. In example when the number of M found in 100 larvae (Zoea-5) is seven, then the MOI value is 0.07 (7%). Observation was done at the day when larvae have been metamorphosys to the megalopa stage mostly started at the day 18–20 DAH by taking water on the rearing media as much as 1 L using scoop then counted the total number of larvae taken and the number of M followed as much as 4 times for each tank.

##### 2.1.4.3 Daily population density (ind/L)

Population density was done by counting the number of larvae in 100 ml of larval rearing culture media. These larvae rearing culture media were sampled using bowl scoop at least 3 times to calculate the mean daily population density.

##### 2.1.4.4 Crab survival and water quality parameters

Survival rate were calculated from i) Zoea 1 to Zoea 5 and M, ii) from Zoea-1 to crablet and iii) from M to crablet stage. Crablet production that is the number of crablets produced at the end of the study for each treatment. Water quality observed includestotal Ammonia Nitrogen (TAN), Nitrite (NO_2_), Total Organic Matter (TOM) and Total Bacteria of *Vibrios* (TBV) were measured on 20 DAH by taking samples of water in each larvae tank and analyzed in the laboratory of water quality and pathology at RIBAFE, Maros.

#### 2.1.5 Data analysis

The data of survival rate, production of crablet and growth were analyzed by parametric test using the Mann-Whitney *U* test and T-test. Normality test used the Shapiro-Wilk test and homogeneity test used the F test. Data analysis used *R-program,* while making graphs used Microsoft Excel Program.

### 2.2 Different microalgae experiments

#### 2.2.1 Experimental design

**Fig. 1:**
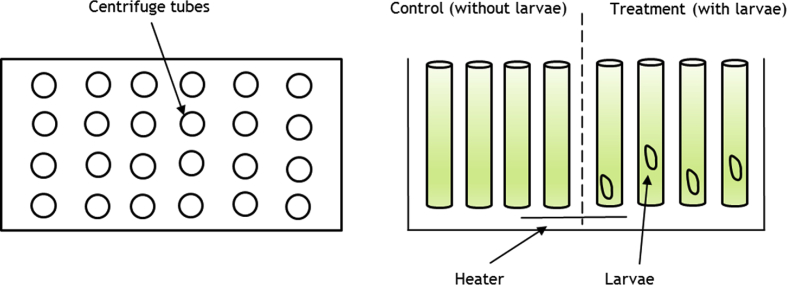
The arrangement of the centrifuge tubes of the ingestion rate experiment by individual *Portunus pelagicus* larvae.

One polystyrene tray with 80 holes was set up in the tank (100L) which the centrifuge tubes 50 ml were placed. In this study, 4 types microalgae were used; *Chaetoceros* sp. only (T1), *Chlorella* sp. only (T2), *Nannocloropsis* sp. only (T3) and *Isochrysis* sp. only (T4). For control, no larvae were placed in the centrifuge tubes. For treatment, one individual larvae were placed in the centrifuge tubes. The polystyrene tray was floated on surface of water to immerse 1/3 bottom part of centrifuge tubes, act as a water bath and one heater was placed at the tank bottom (Fig. 1).

For example in T1: *Chaetoceros* sp. (4 × 10^4^ cells ml^−1^) was filled in 20 pieces of centrifuge tube each at 40 ml (10 tubes each for control and 10 tubes each for treatment). Three time measurement was taken to get initial mean and was recorded as IC_1_ for control and IT_1_ for treatment. One individual crab larvae (Zoea 1, Zoea 2, Zoea 3 and Zoea 4) was taken carefully from master tank culture to prevent rotifer and *Artemia* contaminate the experiment and gently pipette into treatment tubes that state above at 0900 h. Moderate aeration was supplied in each centrifuge tubes. The water heater was used in the water bath culture to control the fluctuation of temperature (28–30 °C). The experiment was conducted from Zoea 1 till Zoea 4 stages. *Artemia* and rotifer were given as standard feed in master tank culture at 0900 h. After 24h, aeration was stopped, all centrifuge tubes were brought to the laboratory for quantification step. The larvae in centrifuge tubes were pippeted out. The density of *Chaetoceros* sp. was determined using Particle Counter machine (SLS 4000). The final densitiy of *Chaetoceros* sp. were labeled as FC_2_ for control and FT_2_ for the treatment. To get the density of *Chaetoceros* sp. ingested by an individually larvae crab after 24h for control AC_3_ and for treatments, AT_3_.

For larvae survival percentage, the initial density of larvae with 20,000 larvae was stocked in 500L of treated seawater cultured tank. After the larvae reach the last day (14 days) of stage (Z4) or initial stages of the megalope stage (M) by observed their morphology, the density of larvae left were calculated as the final amount. The percentage survival (%) at particular treatment was met by dividing the final stoking density with initial stocking density. Each treatment was replicated three times to get the mean value.

Determination of larvae stage index (LSI) of *P. pelagicus* larvae was done according to previous methods [6], [7]. The aeration was stopped for 5 minute; to allow the larvae to aggregate. Larvae were sampled at three different locations (surface, middle and bottom) of the rearing tanks to observe their stages during culture period. The formula to calculate LSI and larval culture techniques was according to the previous published literature [7], [10], [13].

#### 2.2.2 Statistical analysis

Differences between types of microalgae and each larval stage in ingestion rate, survival and Larval Stage Index (LSI) were analyzed by using One-way ANOVA and Tukey HSD Test through application of IBM SPSS Statistics Version 22 software and Microsoft Office Excel 2016.

## Acknowledgments

The present experiment was supported by Aquaculture Research Program DIPA 2016, Research Institute for Brackish-water Aquaculture and Fisheries Extension (RIBAFE), Ministry of Marine Affairs and Fisheries, Indonesia and a grant from the Niche Research Grant Scheme (NRGS) under grant Vote No. 53131 from Ministry of Education, Malaysia. Authors wish to thanks all staff at Research Installation of Marana belongs to Research Institute for Brackish-water Aquaculture and Fisheries Extension, Maros, South Sulawesi, Indonesia. Authors also wish to thanks all staff especially Mr. Mohd Sabri at Hatchery of Institute of Tropical Aquaculture and Fisheries Research, (AKUATROP) for technical assistance through research. The second author is grateful for the financial support received from Ministry of Education, Malaysia. Through the Postgraduate Scholarships (MyBrain15- MyPhD Scholarship).

## Appendix A Supplementary data

The following is the Supplementary data to this article:

Multimedia component 1 Multimedia component 1

## Conflict of interest

The authors declare that they have no known competing financial interests or personal relationships that could have appeared to influence the work reported in this paper.
